# UV protection for young athletes: using participatory program planning to develop a sports schools program

**DOI:** 10.1186/s12199-020-00872-7

**Published:** 2020-08-10

**Authors:** Friederike Stölzel, Michaela Wolff, Vera Fieber, Melanie Glausch, Claudia Wachs, Eckhard Breitbart, Martin Bornhäuser, Nadja Seidel

**Affiliations:** 1 University Hospital Carl Gustav Carus, TU Dresden, Dresden, Germany; 2 National Center of Tumor Diseases (NCT/UCC), Fetscherstraße 74, 01307 Dresden, Germany; 3 Arbeitsgemeinschaft Dermatologische Prävention (ADP), Hamburg, Germany

**Keywords:** UV protection, Skin cancer prevention, Setting intervention, Young athletes, Targeted intervention

## Abstract

**Background:**

The incidence of melanoma increased rapidly throughout the last decades, with overexposure to ultraviolet (UV) radiation being an established risk factor. Due to their intensive sun exposure, many student athletes (SAs) have an increased risk for skin cancer. The Clever in Sun and Shade Program (CSSP) aims at enforcing positive attitudes toward UV protection (UVP) and at supporting sports schools in establishing UVP strategies.

**Methods:**

CSSP was developed in 2019 using participatory program planning (PPP) as well as following WHO recommendations for UVP at schools. After drafting first material, within a PPP groups were conducted at a partner school (convenience sample 1) with students (*n* = 20), teachers (*n* = 5), school administration (*n* = 2), and coaches (*n* = 5). Materials were then adapted. Program acceptance and feasibility were tested at two further schools (convenience sample 2) with PPP groups of students (*n* = 95) and school administration (*n* = 2). Content analyses and descriptive statistics were conducted.

**Results:**

Less than 50% of SAs and coaches of sample 1 expressed positive attitudes toward UVP, less than 10% reported appropriate UVP behavior. By using PPP, program material was adapted to the target groups’ needs, i.e., by including specific barriers and solutions. Only the most accepted video drafts were produced. The majority of SAs of sample 2 (80-86%) used predominantly positive adjectives such as “important” and “positive” to describe the completed videos and the behavior self-check poster.

**Conclusions:**

PPP process has greatly influenced concept and materials of CSSP for sports schools. Integration of future program participants has proven to be an important component in creating a fitting and feasible program. CSSP for sports schools is a program free of charge that enables sports schools to integrate UVP into their daily routine. It will be disseminated in cooperation with *German Olympic Sports Confederation* and *German Cancer Aid* in 2021.

## Background

The incidence of skin cancer has increased substantially throughout the last four decades. In Germany, the incidence for malignant melanoma has more than quintupled since the 1970s. In 2016, around 23,000 people developed malignant melanoma and 230,000 people non-melanotic melanoma for the first time [1]. UV exposure is an important risk factor for skin cancer, including the increasing lifelong dose of UV radiation (cumulative UV exposure), irregular UV exposure (intermittent UV exposure), and sunburn at any age [2, 3]. UV exposure in childhood and adolescence plays an important role in the development of skin cancer [4]. Many health behaviors are formed and established in this period of life, stressing the importance of interventions promoting health behavior to address children and adolescents as target groups [5]. Especially athletes of outdoor sports are exposed to a high dose of UV radiation [6–10]. Mahé et al. [11] found that young outdoor athletes had a significantly higher increase of nevi over 2 years than children who did not exercise outdoors. Young athletes performing outdoor sports can have an increased risk of skin cancer [11, 12]. However, UV protection (UVP) is not regarded well enough by children and adolescents performing outdoor sports [11]. In addition, barriers impede sun protection during training [6].

Therefore, the demand for UVP in young competitive athletes who practice outdoors frequently is high.

Interventions to promote UVP should follow WHO recommendations (e.g., limiting time in the midday sun, using shade wisely, wearing protective clothing, using sunscreen, avoiding sun lamps and tanning parlors) [13]. Further, WHO recommendations state that interventions to promote UVP should be implemented at schools to educate students, to change students’ attitudes and behavior toward UVP [14]. In addition, interventions to promote health should use targeting strategies to effectively promote behavioral change by delivering health messages that are specific to the intended audience [15]. Participatory program planning (PPP) can improve programs by involving the target groups in program development [16]. Due to the high demand of UVP in student athletes (SAs) and the small number of interventions available [6, 12], the authors developed a UV protection program for sports schools. To strengthen future feasibility and acceptance, PPP was used in developing the Clever in Sun and Shade Program (CSSP).

## Methods

CSSP aims at supporting sports schools in establishing UVP strategies and at enforcing positive attitudes toward UVP. The paper describes CSSP development using participatory program planning (PPP) as well as following WHO recommendations for UVP at schools.

Participatory program planning (PPP) includes target group members in the program planning process and changes program development from a top-down to a rather shared approach. The likelihood of a program to succeed will rise with a better fit to individuals’ needs and experiences [16].

In developing CSSP, one part of the PPP group was psychologists and public health professionals organizing the PPP process and preparing program material. To include future program, participants’ views and experiences, another part of the PPP group were potential program participants. Relating to sun protection for young athletes in sports schools, different target groups have to be regarded, starting not only with young athletes but also with coaches, parents, teachers, and school administration.

### The components of participatory program planning

PPP consists of components that, when considered correctly, not only improve program development and implementation but also its future evaluation [16]. The PPP components in regard to CSSP are described in Table 1.

**Table 1:** Program planning components and their application in the Clever in Sun and Shade Program (CSSP)

Component	Purpose	Application in CSSP
1. Participatory input	To include persons that are most affected by a program in the planning	Young athletes, coaches, parents, and teachers have been included in the PPP process to develop CSSP.
2. Stakeholder check-in	To include stakeholders with influence necessary for program implementation	School administrators have been included in the PPP process to develop CSSP.
3. Definition of need and program purpose	To define the need or challenge to be addressed by the program and its purpose	The need for a sun protection program for sports schools arises due to high dosages of ultraviolet (UV) radiation in young athletes, UV radiation being a major risk factor for skin cancer, and an overall low rate of sun protection behavior in this target group. On the organisational level, the purpose of the Clever in Sun and Shade Program is to support sports schools in establishing UV protection strategies. On the individual level, CSSP wants to enforce positive attitudes, intentions, and behavior toward UV protection (UVP).
4. Resource and asset mapping	To determine what resources and assets are available for the program from participants and community	Participants of the future CSSP are young athletes, coaches, parents, teachers, and school administration. Each target group not only presents needs concerning sun protection but also brings along assets and resources that can be of great value to the program. For example, athletes and coaches show a high rate of commitment as well as a high regard for health. Parents contribute to the wish for their child’s well-being whereas teachers and school administration can add the framework and expertise to impart knowledge about sun protection behavior.
5. Ecological environment assessment	To determine the purpose of the program within the context of program participants lives	Schools as an ecological environment can be used to teach students health behavior [14]. Referring to CSSP, the relationships between individuals, for example young athletes among each other, but also in relation to their coaches, teachers, and parents, respectively, are considered on the micro level. The connections between young athletes, coaches, and schools as well as sports leagues and associations are regarded on the meso level.
6. Program design or replication	To determine what type of program might be most appropriate to plan	To achieve positive attitudes and intentions toward sun protection, a method mix as well as a targeted approach was chosen to be used in the development of CSSP. In CSSP, the messages of sun protection are to be communicated using various methods and materials: On one hand, target groups are to be addressed personally via multipliers in school (teachers and trainers via school management, students via teachers, and trainers). On the other hand, posters and videos should reach different target groups through the targeting approach. Targeted messages are based on characteristics of population subgroups to make them relevant to individuals [15].
7. Program theory	To define the explicit components of the program and the assumptions underlying its success	The theory underlying CSSP on the individual level is the health action process approach (HAPA [17];). HAPA is based on the assumption that the adoption, initiation, and maintenance of health behaviors can be described as a process that consists of a motivation phase and a volition phase. Specific cognitions such as perceived self-efficacy, risk perceptions, and outcome expectancies are considered to be important along the process of changing attitudes and should be included in program planning [18, 19]. Regarding risk perception, high UV exposure as a main risk factor for skin cancer is introduced. Outcome expectancies are addressed by account of possible consequences of a high UV exposure, i.e., aging skin and poorer physical performance. The presentation of UVP measures as “easy to implement” target at promoting self-efficacy. Volitional processes are particularly supported by the manual and teaching materials, e.g., addressing the handling of barriers. The behavior self-check poster and integration of UVP into the school year plan promote sustainability of UVP behavior. Based on social learning theory [20, 21], for all students a former canoe world champion as well as student athletes of grade 9 to 11 were chosen as role models for the videos. For adults, the ambassador for skin cancer prevention of German Cancer Aid, a coach, a teacher and one parent were chosen. On the organizational level, CSSP uses the setting approach. Understanding school as a setting [22], CSSP is not only designed to influence individual determinants of behavior among specific target groups (e.g., students, teachers) but also school processes and structures. CSSP introduces a manual holding wording for UVP policy and further materials available to implement UVP into school structures.
8. Program goals	To create agreed upon program goals and objectives that define the purpose of the program	On the organizational level, CSSP aims at supporting sports schools in establishing UV-protection strategies: 100% of schools that are awarded to be a CSSP school should establish a sun protection strategy including informing all target groups and supporting UVP-measurements. On the individual level, CSSP wants to enforce positive attitudes and intentions toward UV protection: 70% of students/young athletes, coaches and teachers of schools that are awarded to be a CSSP school should know about UVP. 70% of parents of students in schools that are awarded to be a CSSP should know about UVP. 70% of students/young athletes, coaches, teachers, and parents of schools that are awarded to be a CSSP school should rate adherence to UVP in training and competition as being important. 70% of students/young athletes of schools that are awarded to be a CSSP school should apply UVP as much as possible in regard to the respective sport disciplines.
9. Policy considerations	To identify the larger macro structures such as funding and outside support	Policies that are regarded in developing CSSP concern students’ health, occupational safety, guidelines of the national cancer initiative, and “healthy school” (*Gesunde Schule*). For example, the resolution of the German Conference of Ministers of Culture on “Health Promotion and Prevention in Schools” recommends using the setting approach for school interventions. Interventions should also be circumstance and behavior oriented and designed to be participatory [23].
10. Evaluation plan	To develop a plan for an on-going participatory evaluation of the program as well as a time line for external, outcome evaluations	In developing CSSP an evaluation approach has been considered early: Data collection on program acceptance and feasibility has been included into the PPP process. Summative evaluation will follow in a subsequent study.

### The process of participatory material development

Before target groups were included in program development, a concept for design and materials of CSSP was drafted based on purpose and goals as well as program theory (Table 1).

For developing a UV-protection program for sports schools via the PPP approach, a convenience sample of three schools was chosen out of the 43 “elite school of sports” that is supported by the German Olympic Sports Confederation (DOSB, *Deutscher Olympischer Sportbund*).

At partner school 1 (*Sportgymnasium* Neubrandenburg; Latitude: 53.5678292, Longitude: 13.2779269), 498 students are taught by 41 teachers plus 9 sports teachers/coaches. At partner school 2 (*Sportoberschule* Dresden; Latitude: 51.0504088, Longitude: 13.7372621), 240 students are taught by 17 teachers plus 8 sports teachers. At partner school 3 (*Sportgymnasium* Dresden, coordinates see above), 482 students are taught by 53 teachers plus 9 sports teachers.

For further convenience sampling in each school, the school administration defined eligible participants out of the target groups. In sample 1 and 2, the school administration decided based on their schedules which students, teachers, and coaches could take part in the PPP process. In sample 2, in addition, the principal investigators required every second grade from 5 to 12 should participate. Therefore, in partner school 2, the school administrator selected two classes out of grade 7 and 9, and in partner school 3, one class of grade five and students of grade 12 were chosen. Further students, coaches and teachers were chosen to receive a behavior questionnaire whose results are not reported here. In both samples, no parents took part.

The PPP process in developing CSSP was structured into the following four steps.

#### Step 1: drafting program concept and material

In preparation, literature research was carried out on sun protection during sports, sun protection programs at sports schools as well as on UVP and barriers toward UVP among students and coaches. In succession, a first concept for CSSP was developed. Considering that CSSP should be implemented in sports schools, feasibility, low costs, low manpower requirements as well as low expenditure of time were regarded closely. Thus, the authors decided for digital program materials conveying sun protection behavior messages, next to posters and information for school administration about possibilities to sustainably implement sun protection into school structures and processes. Among others, the wording for a paragraph was prepared to anchor sun protection in the house rules of the schools. To encourage schools to participate in CSSP, the Clever in Sun and Shade award along with its criteria was included into program planning. Ideas for digital program materials included short videos targeting teachers, coaches, parents, and students. Videos drafts for the latter were designed to link UVP messages to values and attitudes common to athletes, such as being performance-oriented and assertive [24–26]. Additional ideas for videos included slapstick comedy tailored by sports disciplines to target students.

#### Step 2: formative evaluation of program concept and material drafts based on PPP group meetings in partner school 1 (sample 1)

Program concepts and materials were evaluated at *Sportgymnasium* Neubrandenburg (partner school 1). In January 2019, three PPP group meetings were conducted with members of the potential participating group of students (*n* = 20), teachers (*n* = 5), and coaches (*n* = 5). School administration (*n* = 2) were also part of the meetings as representative stakeholders. Participating teachers were teachers of biology, German, physics, art, and social studies. The need for UVP in young athletes as well as the purpose of a UVP program for sports schools were presented to group participants and in succession discussed, also in regard to survey present UVP behavior as well as barriers for UVP in training and competition. Within the meeting with coaches, options for promoting sun protection during training were discussed. Within the meeting with teachers, options for addressing sun protection as a topic in class were highlighted. First drafts of CSSP materials were then assessed in all PPP group meetings, especially focusing on acceptance. Students answered a short questionnaire about the 14 video drafts: acceptance was surveyed with the question “How do you like the idea for the video?” and measured with a four-point Smiley Face Likert scale (1 = strongly disagree, 2 = disagree, 3 = agree, 4 = strongly agree). Identification with the videos was asked with the question “What video can you identify with?”. Students could then apply adhesive dots at printed drafts of the videos according to their identification with the video idea. Observations as well as the spoken word of the PPP group meetings and the interviews were recorded. UVP behavior-frequencies from students and coaches were subsequently analyzed. Based on the four-point Smiley Face Likert scale, frequencies of acceptance were analyzed as well as medians and interquartile ranges for each video draft targeting SAs. Regarding identification with program material, frequencies of dots adhered to printed drafts of videos targeting SAs were also evaluated. Descriptive analysis was conducted with Microsoft Excel 2016. Content analysis of the PPP group meetings’ records were conducted and opinions and statements of the participants were extracted.

#### Step 3: adaption of program material and video production

Based on the results of PPP-group meetings in sample 1, posters, drafts for the videos, and further material were adapted. The video spots were produced in May and June 2019, at partner school 1, including students (*n* = 21), trainers (*n* = 2), one teacher, and one parent as actors.

#### Step 4: formative evaluation of CSSP based on PPP group meetings in partner school 2 and 3 (sample 2)

In September 2019, CSSP was piloted at *Sportoberschule* Dresden (partner school 2) and *Sportgymnasium* Dresden (partner school 3) which are also supported as “elite schools of sports” by DOSB. Feasibility was examined via structured interviews with the two school principals before and after conducting CSSP. In four PPP group meetings with students of grade 5, 7, 9, and 12 (*n* = 95), UVP behavior and acceptance of videos and posters were surveyed. All students were asked to indicate their identification with the videos, applying up to five adhesive dots at printed images of the five produced videos answering to the question “What video can you identify with?”. Regarding the acceptance of videos and poster, SAs were asked to select adjectives to describe their impressions, e.g., “important,” “positive” “moving” “boring” “irritating.” Three out of the four PPP groups (students of grade 7, 9, and 12; *n* = 71) selected up to 5 out of 15 positive and 11 negative adjectives for the videos as well as the behavior-check poster. Observations and interviews in partner schools 2 and 3 were also recorded. In a first step of data analysis, frequencies of selected adjectives were calculated. Frequencies are depicted in word clouds. In a second step, it was examined whether students chose more positive than negative adjectives. Therefore, the differences (d_k_i_; i = index material = 1-6) between positive adjectives (x_k_i_) and negative adjectives (y_k_i_) were calculated for each video (k_1_-k_5_) and the poster (k_6_). Based on the differences, a dichotomous variable “predominantly positive adjectives selected” was calculated for each video and for the poster with the values “yes” (p_k_i_ = 1; if d_k_i_>0; if x_k_i_ > y_k_i_) and “no” (p_k_i_ = 0 if d_k_i_ ≤ 0; if x_k_i_ ≤ y_k_i_). Subsequently a new variable was calculated, summarizing results of the videos. The variable indicates whether more than half of all rated videos were described with predominantly positive adjectives (“yes” if (∑p_k_i_/2)>2; “no” if (∑p_k_i_/2) ≤ 2; *i* = 1-5). Identification with videos targeting students was analyzed by calculating absolute and relative frequencies of adhered dots. Descriptive analysis was conducted with Microsoft Excel 2016. Analog to content analysis in sample 1, records of PPP group meetings were analyzed by extracting opinions and statements.

## Results

### Application of the components of participatory program planning

The application of the PPP components introduced by Nichols [16] in regard to CSSP is described in detail in Table 1. With regard to PPP components, CSSP was able to regard all components starting not only with “participatory input” (1st component) via including the intended target groups but also with “stakeholder check-in” (2nd component) by inviting school administration to take part in the planning process. The definition of need and program purpose (3rd component) arises out of the increasing incidence of melanoma and nonmelanoma skin cancer throughout the last decades representing a growing health risk. Within the group of children and adolescents, student athletes form a subgroup especially at risk for an overexposure to UV radiation due to their frequent outdoor training sessions as well as their oftentimes insufficient UV protection behavior [11]. “Resource and asset mapping” (4th component) helped to identify what resources from future CSSP participants are available for the program, such as student athletes, coaches but also parents showing a high regard for health. Based on “Ecological environment assessment” (5th component), the program was designed to be integrated into sports school processes as one of the main areas of students’, coaches’, and teachers’ lives. “Program design” (6th component) is realized in CSSP via addressing future program participants with targeted UVP messages that are conveyed with various materials such as videos, posters, and emails.

The theories underlying CSSP (7th component) are the health action process approach (HAPA, [17]) and Social Learning Theory [20, 21] on the individual level, and the setting approach on the organizational level setting [22]. “Program goals” (8th component) supported operationalizing CSSP objectives throughout the PPP process in 2019. The goals focus on sports schools that have implemented the program and are awarded as Clever in Sun and Shade. With implementing CSSP, schools should have a sun protection strategy. On the individual level, knowledge about UVP behavior and positive attitudes toward UVP should be present in 70% of the target groups (student athletes, coaches, teachers, and parents). Furthermore, 70% of the target groups should show a proper UVP behavior. “Policy considerations” (9th component) are directly integrated into designing CSSP, with corresponding guidelines and policies from national cancer and healthy schools initiatives supporting a targeted and participatory approach [23]. Specific program goals are mandatory for the “Evaluation plan” (10th component). The defined CSSP program goals address summative evaluation that the authors are planning to conduct in a subsequent study in 2021. Formative evaluation on acceptance and feasibility has been included into the PPP process. Ratings on acceptance of and identification with videos and behavior self-check poster of the participating student athletes have been used to modify program material and to adapt it to the needs of this target group.

### Description of the process of participatory material development

#### Step 1: drafting program concept and material

Purpose of CSSP on the organizational level is to support sports schools in establishing UV protection strategies. CSSPs’ conceptual design is a project kit free of charge that enables easy implementation of UVP into sports schools’ daily routine, providing easy-to-use instructions for activities and materials. In the process, schools can document their participation and apply for the Clever in Sun and Shade award. On the individual level, CSSP wants to enforce positive attitudes, intentions, and behavior toward UV protection.

Including organizational and individual level, CSSP material was drafted, covering wording to incorporate UVP into house rules, award criteria, a behavior self-check poster, and short videos on UVP targeting teachers, coaches, parents, and students. Drafts of CSSP material are presented in Table 2.

**Table 2:**
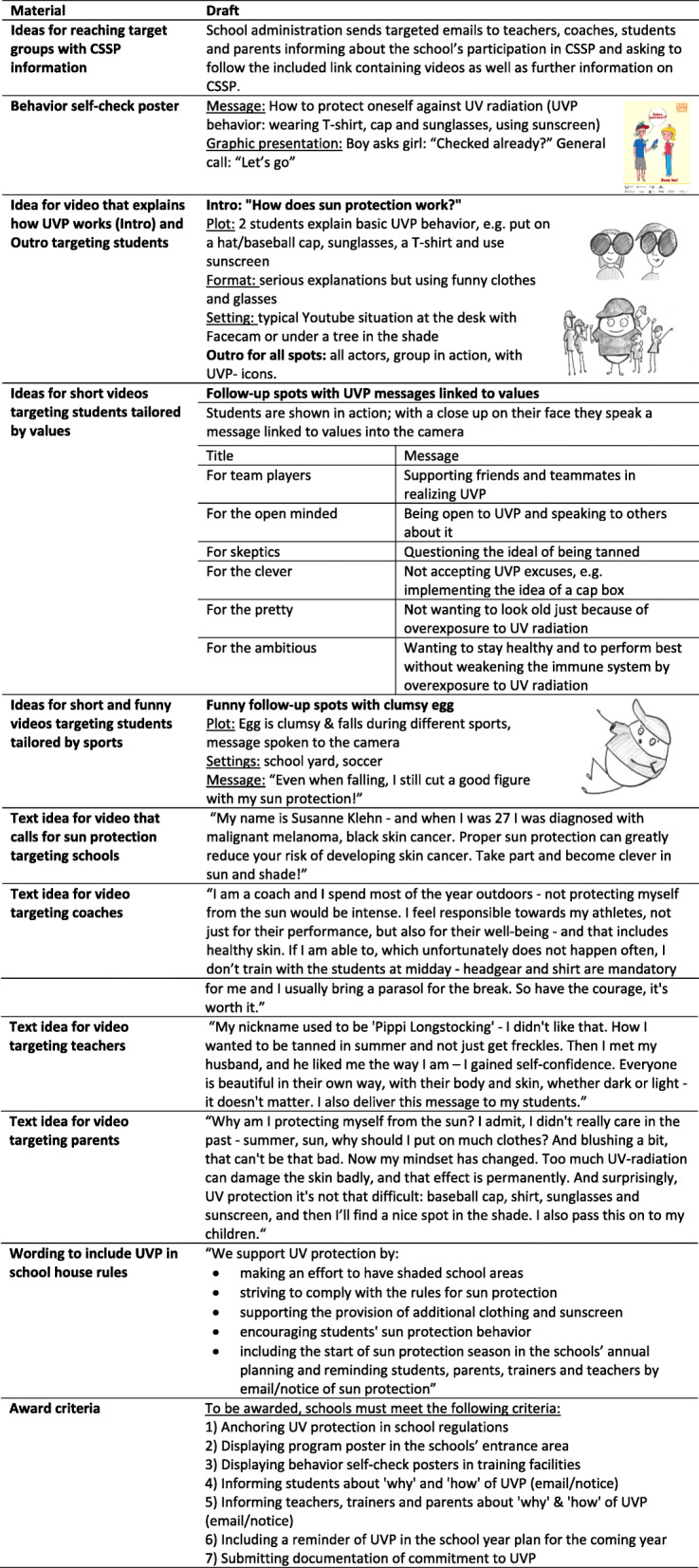
Drafts of CSSP material in January 2019

#### Steps 2 and 3: formative evaluation of program concept and material drafts based on PPP group meetings in partner school 1 (sample 1) and adaption of program material and video production

To illustrate the changes made following the PPP group meetings in partner school 1, the results of steps 2 and 3 will be presented together. First, results in relation to attitudes toward UVP, UVP behavior, and barriers to UVP will be reported, followed by reports on adaption of program material.

In January 2019, three PPP group meetings were conducted at *Sportgymnasium* Neubrandenburg (sample 1). Participants of the PPP group meetings were members of the potential participating group of students (*n* = 20; 9 male and 11 female), teachers (*n* = 5; 2 male, 3 female), and coaches (*n* = 5; 3 male, 2 female) as well as school administration (female) and social worker (male). Teachers of biology, art, physics, English, and German participated. Students were in grade 9 (*n* = 3), 10 (*n* = 6), and 11 (*n* = 11). Among the participating students and coaches were canoeists, soccer players, track and field athletes, and triathletes.

##### Attitudes, UVP behavior, and barriers

Being asked about their attitudes toward UVP, 4 of 5 coaches (80%) reported a positive attitude, emphasizing statements such as “it’s important to regard UVP.” One coach described sun protection as “more than just relevant.” In addition, another coach reported a negative attitude toward tanned skin, because it “draws so much energy” that athletes need for their performances. When looking into actual UVP behavior, no coach reported adequate UVP behavior according to recommendations (avoid midday sun, shade, shirt, hat, sunglasses, sunscreen) for themselves as well as not emphasizing UVP behavior for their athletes. Some coaches stated their responsibility toward their athletes whereas others emphasized individual responsibility of SAs.

Of 20 SAs taking part in the PPP groups of sample 1, less than 50% agreed to the importance of UVP and that their attitudes toward UVP are positive. Regarding their present UVP behavior, only 10% of the students (*n* = 2) reported to meet UVP recommendations. Track and field athletes reported that, if anything, they protect themselves with sunscreen in the stadium, whereas baseball caps and sunglasses are rarely used. SAs also report a desire for tanned skin as well as a use of UVP only when the weather is especially hot and sunny. One student said that he only protects himself “when the first sunburn has arrived.”

Student athletes as well as coaches specified barriers for UVP in training and competition such as rules for clothing (e.g., no hats for soccer players), predetermined times for training, obstruction by UVP in performing (e.g., slipping off the oars/rudders when sun lotion has been applied), and the lack of shade at training facilities (e.g., soccer field, stadium). Ideas to overcome barriers have been mentioned by the coaches, for example, rubbing hands with sand after applying sunscreen, adapting time, and location for training if possible, e.g., for triathletes, using rules to support UVP behavior (shirt is mandatory, applying sunscreen in the changing room). Students affirmed their willingness to accept behavioral rules set by their coaches.

##### Adaption of the behavior self-check poster

**Fig. 1:**
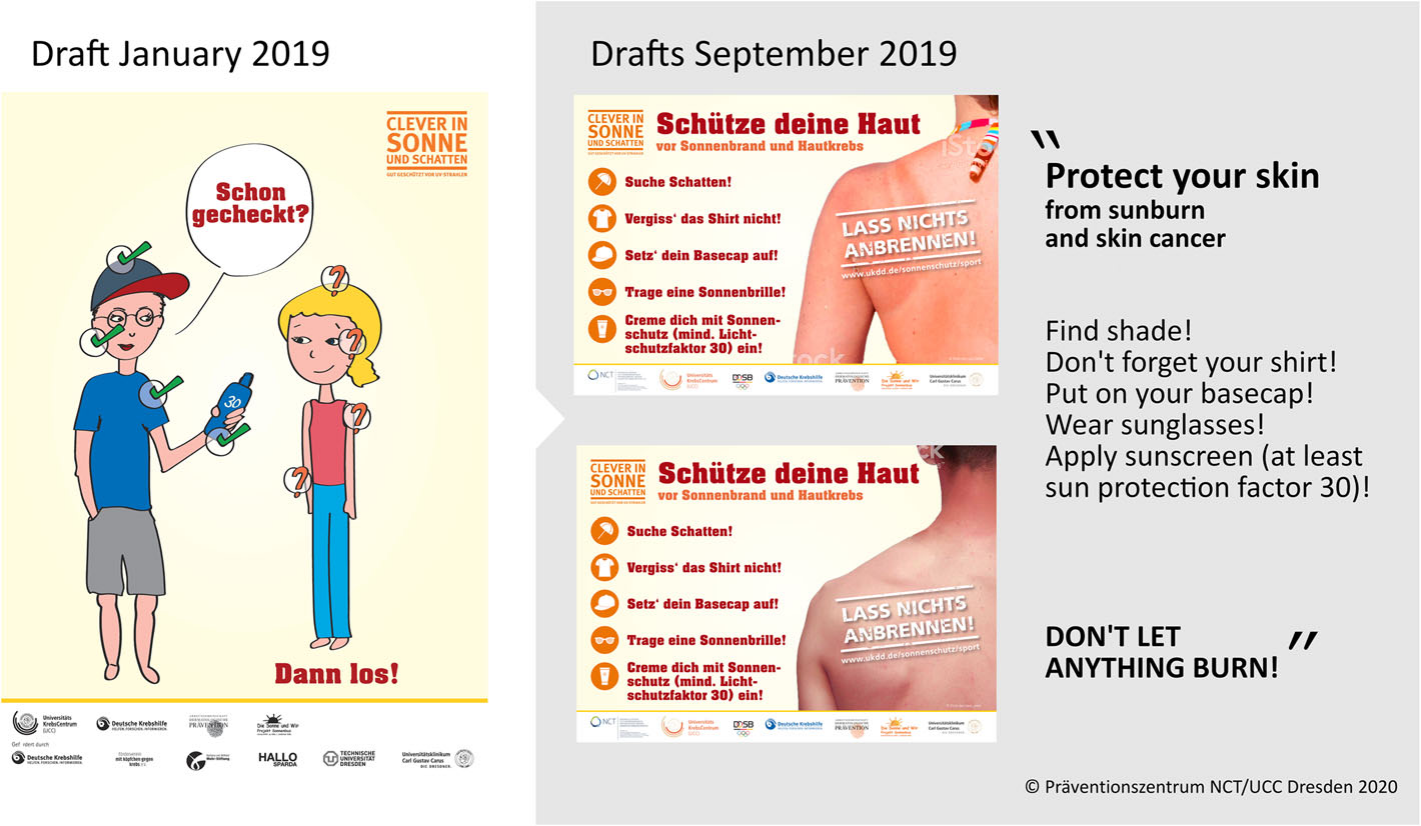
Adaption of the behavior self-check poster draft after PPP group meetings in partner school 1

Students of sample 1 examined the behavior self-check poster and required more directive UVP messages (e.g., “Don’t forget your shirt!”) instead of questions as well as images of negative consequences of missing UVP like skin cancer or sunburn on the poster. They also stated to prefer photos to a drawing and icons of UVP, especially if they have recognition value, to ticks or thumbs up/thumbs down. The poster has then been adapted to the needs of SAs and was adjusted by gender to target females and males (Fig. 1).

##### Adaption of videos targeting student athletes

The videos targeting SAs were predominantly designed to link UVP messages to values and attitudes common to athletes, such as being performance-oriented and assertive. The videos passed a selective process by students in the PPP groups. Acceptance is reported in Table 3, showing the lowest acceptance rates for the video ideas “For the open minded,” ‘For skeptics’ and “Clumsy egg playing football”.

**Table 3:** Acceptance of different ideas for videos targeting students (*n* = 15)

Video idea	Median*	Interquartile range^a^
For the open minded	2	1
For team players	3	0
For skeptics	2.5	1
For the clever	3	2
For the pretty	3	1
For the ambitious	4	0
Clumsy egg in the schoolyard	2	0
Clumsy egg playing football	3	2

^a^Data based on four-point Smiley Face Likert scale (1 = strongly disagree, 2 = disagree, 3 = agree, 4 = strongly agree)

**Fig. 2:**
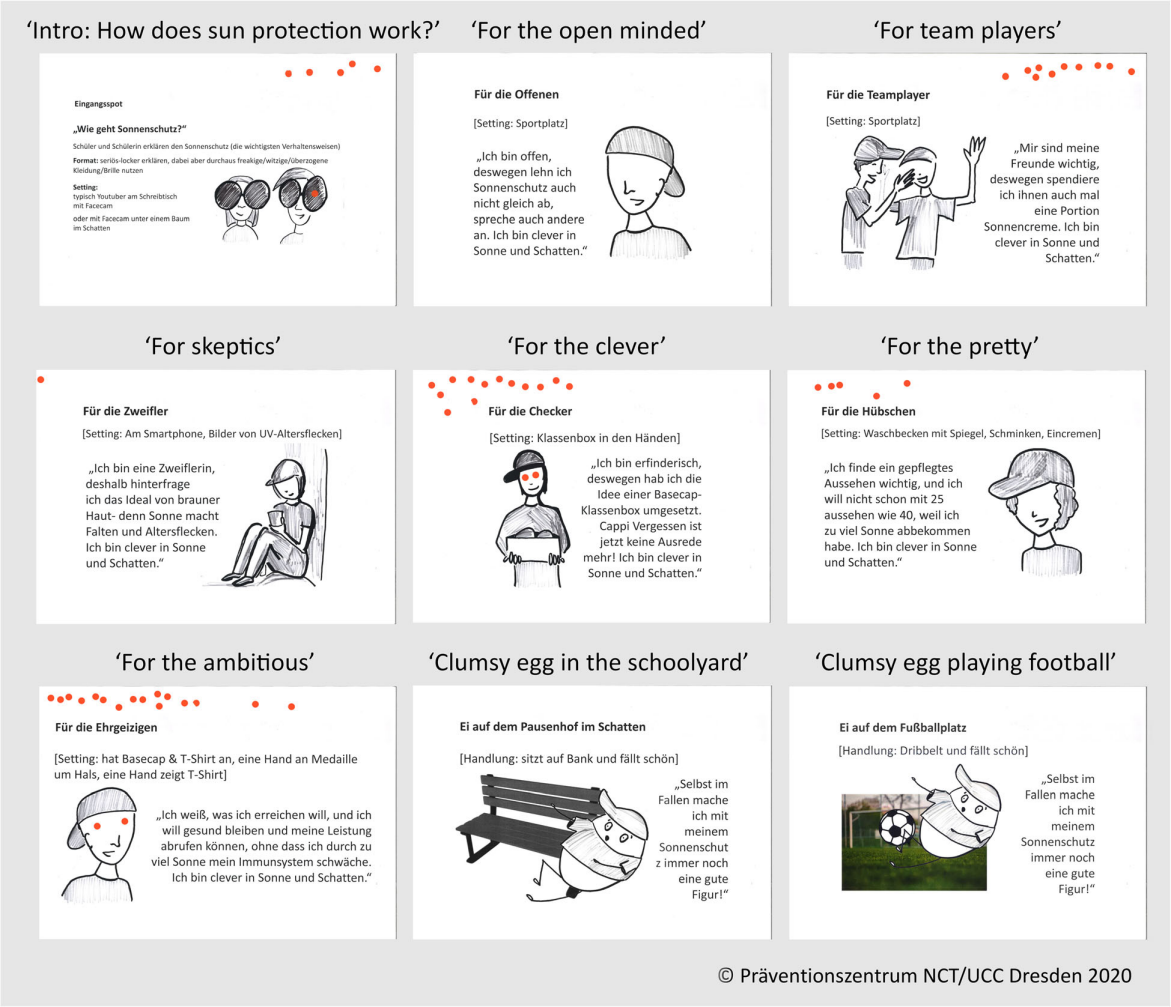
Adhered dots on selection of printed drafts of video ideas for student athletes

Using adhered dots on printed drafts of video ideas for student athletes, the lowest identification was measured for the videos “For the open minded,” “For skeptics,” “Clumsy egg in the schoolyard,” and “Clumsy egg playing football” (Fig. 2). Combining the results of both steps, the video ideas “For the open minded,” “For skeptics,” and the slapstick drafts “Clumsy egg in the schoolyard” and “Clumsy egg playing football” were dismissed.

##### Adaption of material targeting coaches, teachers and school administration

Relating to video ideas for their target group, coaches, teachers, and school administration favored spots with a short spoken message but many positive and strong pictures emphasizing the importance of UVP. They suggested that the videos show parents, teachers, and trainers in everyday situations. Authenticity was particularly important to coaches. The drafted text within the video for coaches (Table 2) was “too perfect, too naive and too complicated” for the coaches of sample 1. Like the students, coaches also wanted a clear message as to “what to do.” Regarding the video for teachers, the drafted text for the teacher was assessed to be “too naive and lengthy.” Videos for coaches, teachers, and parents were then adapted to show authentic role models for the target groups in everyday life. In the video targeting coaches, attention was paid particularly to barriers in training and that coaches can only act within certain frame conditions (e.g., fixed training schedules). The drafts of all videos targeting adults were adapted providing clear messages on how to support young athletes’ sun protection. In addition, an explanation of sun protection by the ambassador of the German Cancer Aid for skin cancer prevention was planned for all videos targeting adults. In a new video draft, Paul Mittelstedt, a former canoe world champion, stresses as a role model the importance of UVP and invites sports schools to take part in CSSP.

Relating to UVP policy, school administration and teachers participating in the PPP groups proposed for CSSP to offer a wording for a sun protection policy. The sun protection policy should not be included in the house rules, but in the school program. The school principal supported that “one should formulate a general wording, but the school program would be a better fit.” In addition, she pointed out that such a policy within the school program could only be used for school and physical education, but not for club training. At partner school 1, club training has its own structures and policies at partner school 1. Regarding the award criteria, school administration, teachers, and coaches agreed with the criteria list shown in Table 2 and had no suggestions for changes.

School administration and teachers of sample 1 expressed a need for supportive offers to educate students about UVP. They evaluated the different options proposed for addressing sun protection as a topic in class and for integrating it into school processes. A teacher said that teaching materials are more acceptable if concepts do not have a “predetermined roadmap” so that schools and teachers can “plan independently.” In addition, it was proposed to establish a platform with a collection of teaching materials. Teachers and school administration mentioned biology classes, sports theory classes, physics classes to address, UVP as well as reading material in language and art classes on the topic of tanned skin in relation to the ideal of beauty. It was particularly important for teachers to inform parents about the importance of sun protection, presentations for parent-teacher conferences, and leaflets on UVP were discussed.

Based on the PPP group meetings, a manual was drafted to inform school administration, teachers, and coaches about the need for sun protection, recommendations on UVP and proposals of UVP-measures at school, in classes and training. Three scopes to implement UVP at schools were covered: scope 1 “first steps” (applying posters and spreading video messages to teachers, coaches, students, and parents), scope 2 “planning a UVP strategy” and scope 3 “reactivating UVP in the following year.” The manual also describes barriers according to literature and PPP group discussions. Furthermore, a website [27] was created to inform about CSSP.

**Fig. 3:**
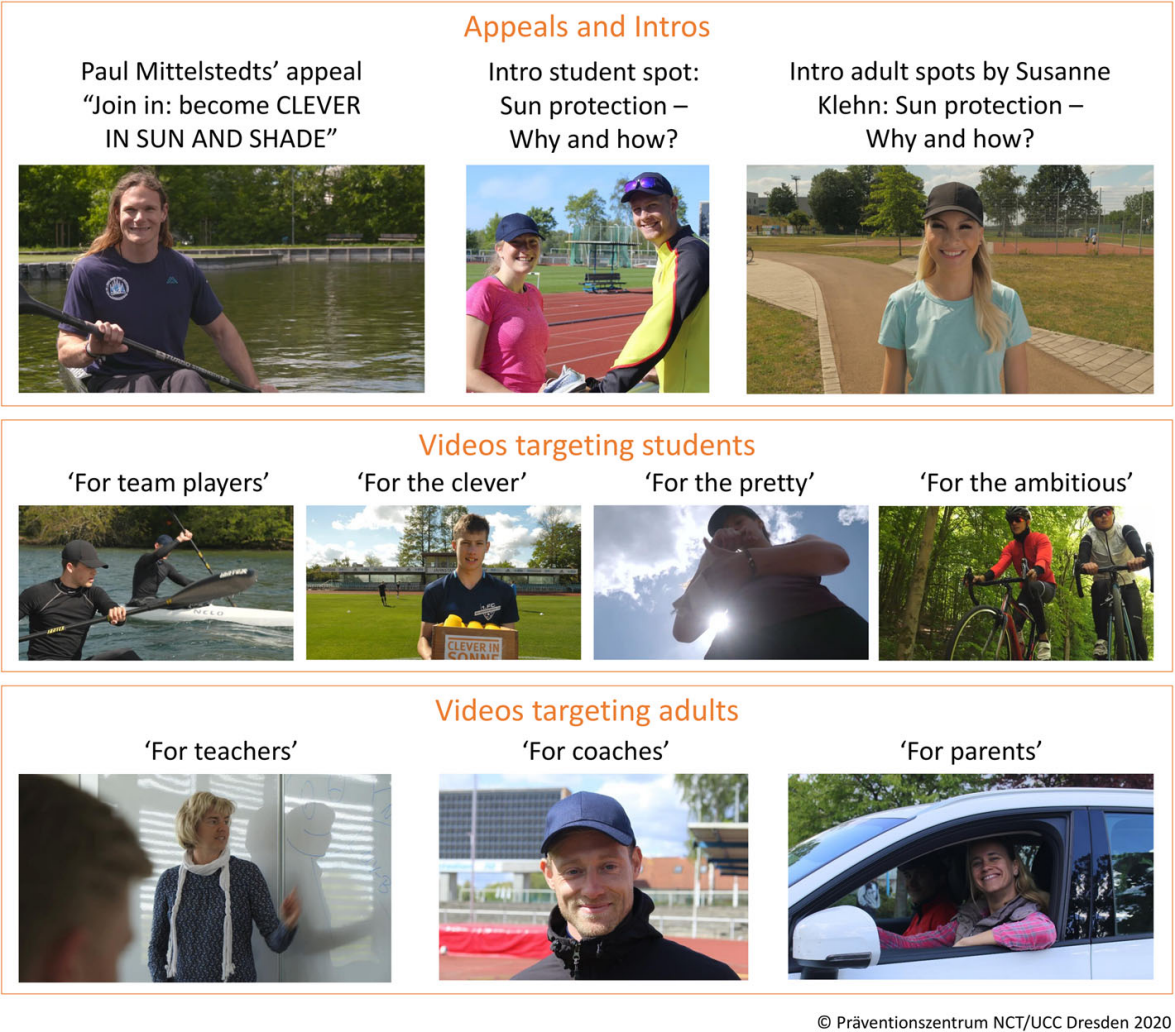
Videos produced in May and June 2019

The videos were produced in May 2019 at partner school 1 (Fig. 3). In June 2019, an additional program video was produced in Dresden with the ambassador for skin cancer prevention of the German Cancer Aid and young athletes (*n* = 12). In preparation of step four, the first versions of the videos were uploaded on the project website.

#### Step 4: formative evaluation of CSSP based on PPP group meetings in partner schools 2 and 3 (sample 2)

The fourth PPP step comprised piloting CSSP at *Sportoberschule* and *Sportgymnasium* Dresden in September 2019. The interviews were conducted with a male (partner school 2) and female (partner school 3) principal. The PPP groups (*n* = 95) with students of grades 5, 7, 9, and 12 consisted of 58% male students. Fifty-seven percent of the students performed soccer, triathlon, track and field, canoeing, rowing, and tennis (*n* = 54), 43% (*n* = 41) performed indoor sports such as speed skating but also conducted endurance training outdoors.

##### Acceptance of program materials and identification with videos of students

Data on acceptance of each video targeting SAs and the behavior self-check poster from students (*n* = 64; grade 7, 9, 12) is shown in Table 4. The summarized variable for the videos shows that 80% of these students described more than half of the videos with predominantly positive adjectives. Eighty-five percent of the students selected predominantly positive adjectives for the behavior self-check poster. Acceptance varied with grade level: in class 9, acceptance was highest (88% for the videos; 100% for the poster), followed by class 12 (85%; 88%) and class 7 (76%; 71%).

**Table 4:** Acceptance of materials indicated by frequencies of students that predominately selected positive adjectives (*n* = 64)

Video spots and poster	Grade 7 (*n* = 21)	Grade 9 (*n* = 16)	Grade 12 (*n* = 28)	Total (*n* = 64)
	*n* (%)	*n* (%)	*n* (%)	*n* (%)
Intro: UVP—why and how?	14 (66.7)	7 (43.8)	23 (88.5)	44 (69.8)
For the ambitious	16 (76.2)	10 (62.5)	15 (53.6)	41 (63.1)
For the clever	15 (71.4)	15 (93.8)	21 (77.8)	51 (79.7)
For team players	19 (90.5)	13 (86.7)	26 (100.0)	58 (93.5)
For the pretty	11 (52.4)	9 (60.0)	16 (61.5)	36 (58.1)
Behavior self-check poster	15 (71.4)	15 (100.0)	21 (87.5)	51 (85.0)

**Fig. 4:**
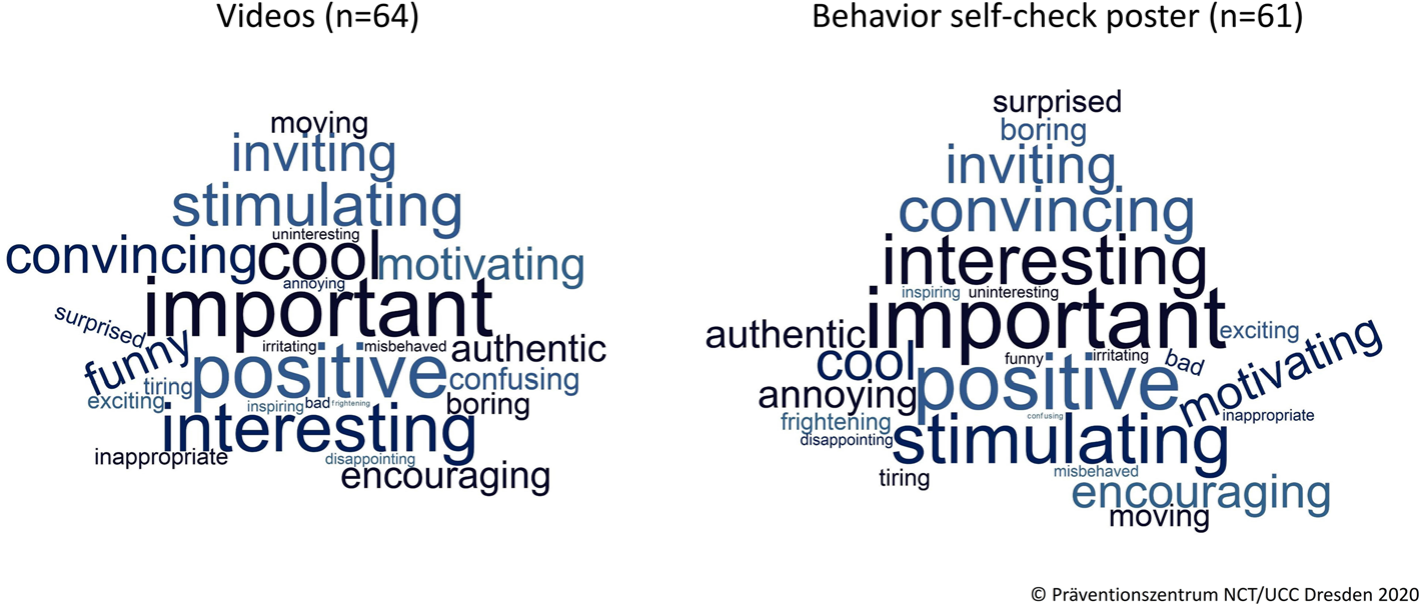
Acceptance of materials indicated by selected adjectives from students (*n* = 64)

Figure 4 shows acceptance of materials from students (grade 7, 9, 12) based on adjectives chosen for videos and behavior self-check poster as word clouds, word size represents frequency of selected adjectives. The videos were most frequently rated as “important” (41%), “positive” (31%), “cool” (30%), “interesting” (20%), “stimulating,” and “inviting” (each 17%). The behavior self-check poster was most frequently rated as “important” (59%), “positive” (51%), “interesting” (36%), “stimulating” (30%), “inviting” “cool” and “convincing” (each 21%).

Most of the students of all 4 PPP groups (*n* = 95) could identify themselves with the video “For team players,” followed by “For the clever” and “For the ambitious,” varying with class level (Table 5).

**Table 5:** Identification of students with videos indicated by frequencies of adhered dots by grade (*n* = 95)

Video spots	Grade 5 (*n* = 24)	Grade 7 (*n* = 22)	Grade 9 (*n* = 16)	Grade 12 (*n* = 33)	Total (*n* = 95)
	*n* (%)	*n* (%)	*n* (%)	*n* (%)	*n* (%)
Intro: UVP—why and how?	6 (7.3)	4 (5.5)	3 (4.2)	16 (18.0)	29 (9.2)
For the ambitious	26 (31.7)	22 (30.1)	16 (22.5)	7 (7.9)	71 (22.5)
For the clever	12 (14.6)	20 (27.4)	27 (38.0)	15 (16.9)	74 (23.5)
For team players	19 (23.2)	21 (28.8)	18 (25.4)	43 (48.3)	101 (32.1)
For the pretty	19 (23.2)	6 (8.2)	7 (9.9)	8 (9.0)	40 (12.7)
Total	82 (100.0)	73 (100.0)	71 (100.0)	89 (100.0)	315 (100.0)

##### Program feasibility and acceptance of school administrations

Feasibility was examined via interviews with the two school principals before and after conducting CSSP. Partner schools 2 and 3 carried out the “first steps” (CSSP scope 1) in September 2019. Program posters were placed in the schools’ entrance halls and in the cafeterias. The behavior self-check posters were placed in corridors, locker rooms, and above washbasins in the student restrooms. Both schools informed about participating in CSSP on their website. In partner school 2, the principal informed teachers and coaches personally or with a notice including a reference to the video messages about CSSP and UVP. Students were informed by teachers or written notices and invited to watch the CSSP videos. Furthermore, teachers showed video messages in class. Partner school 3 planned to inform students, teachers, and coaches by email, but due to technical difficulties in the mailing system, the target groups were informed with written notices.

Both principals gave positive feedback on CSSP after implementation of the program. They assessed UVP to be an important topic for sports schools, the program easy to carry out, and well-thought-out materials. They reported that spots targeting parents, teachers, and coaches were shown to members of the respective target groups and were appraised very positively. Administrations of both schools explained their plans for a sun protection strategy (CSSP scope 2): partner school 2 included UVP in the school’s prevention concept. To sensitize students to UVP behavior, the school also plans to establish UVP as a topic in every grade. One idea was to implement a lesson on UVP in biology, 5th and 6th grade, and to take up further aspects in later grades (e.g., ideal of beauty of tanned skin). Partner school 3 wanted to gather information about funding for measures to create additional shade such as solar panels for the schoolyard.

Both principals stressed the importance of involving parents into CSSP and to provide information for them on UVP, e.g. with material for parent-teacher conferences. They also planned to pursue CSSP the following year (CSSP scope 3).

##### Adaption of program material

To improve the recognition of the sunburn pictured on the behavior self-check poster, the color contrast was set higher due to the students’ comments of sample 2.

Furthermore, the manual was enhanced, taking results of CSSP pilot testing in partner schools 2 and 3 as well as the accompanying PPP group meetings and interviews with the principals into account. Chapters on how to conduct CSSP were adapted, including the following:

- Written notices as ways to inform about the program and the video messages
- Wording for a sun protection policy within the school program
- Incorporation of additional organizational measures for schools
- Incorporation of additional measures for coaches in training and competition
- Incorporation of additional proposals for covering UVP as a topic in class
- Incorporation of additional information on handling UVP barriers

**Fig. 5:**
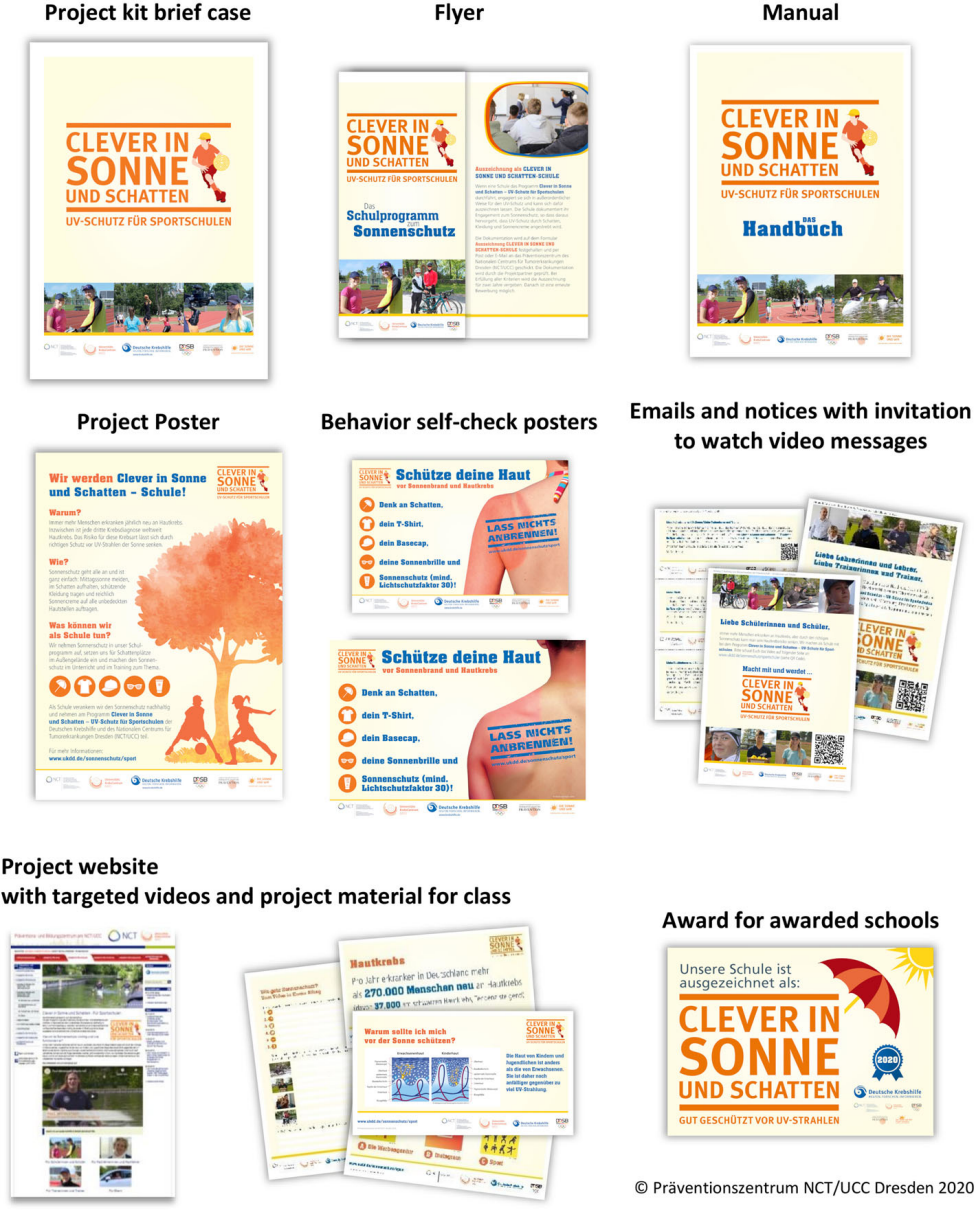
Completed CSSP material

The changes made resulted in additional material such as handouts and presentations for parent-teacher conferences, and led to the current CSSP material package displayed in Fig. 5.

## Discussion

This manuscript reports the development of the Clever in Sun and Shade Program for sports schools (CSSP) that aims at supporting schools in establishing UV protection (UVP) strategies and to enforce positive attitudes toward UVP in student athletes, coaches, teachers, parents, and school administration. In line with the definition of health-promoting programs [28], CSSP wants to empower individual participants to improve their UVP behavior as well as support schools and training facilities in creating UVP friendly environments and processes.

To effectively address these target groups, the authors followed WHO recommendation to place a UVP program at the school setting [14]. In our study, all target groups welcomed school as an appropriate program setting, since sports schools show a special need for adequate UVP. Besides, school as a setting provides many opportunities to inform about UVP. Aiming at increasing the chances of creating a feasible and accepted program, participatory program planning (PPP) was applied as approach which involves target groups in program development [16]. De Castro-Maqueda et al. [8] and Mahé et al. [11] describe SAs and adolescent sport competitors as a risk group for overexposure to UV radiation, since they frequently train outdoors in combination with oftentimes insufficient UVP behavior. In addition, UV protection is sometimes impeded by type of sports and competition rules [8, 9]. The need for sun protection measures is underlined by our finding that only 10% of SAs and none of the coaches of sample 1 reported to meet UVP recommendations. De Castro-Maqueda et al. [29] also report insufficient UV protection habits among physical education teachers who oftentimes spend a lot of time outdoors and whose exposure to UV radiation is significantly elevated compared to other teachers. In CSSP, video spots address coaches, teachers, and parents, but most of the program material is aimed at students. Since the exposure to ultraviolet radiation of coaches is very high [29], they should be addressed even more in the program.

Regarding relevant resources and assets as well as the ecological environment, research has shown that coaches and parents play an essential role in the development of young athletes and in maintaining athletes’ health [30, 31]. In the PPP group discussions, it was observed, that some coaches stated their responsibility toward their athletes, and students affirmed their willingness to accept behavioral rules set by their coaches. In contrast, other coaches emphasized the individual responsibility of SAs. Hence, CSSP wants to support coaches in their role to influence SAs’ UVP behavior. CSSP program was designed to be integrated into sports school processes as one of the main areas of students’, coaches’, and teachers’ lives. Being an important part in student athletes’ but also schools’ lives [31], parents are also addressed by CSSP.

The “participatory input” as well as the “stakeholder check-in” provided valuable input to enhance acceptance and feasibility of the program. One coach in sample 1 underlined the need for athletes to show good performances and therefore to stay healthy, which can be used as a strong argument for a UVP strategy in SAs and coaches. The statement of tanned skin hindering optimal performance (“it draws so much energy”) is in line with the finding that UV radiation suppresses the immune system in multiple ways [32]. Since intense exercise itself can have adverse effects on the immune system, especially endurance exercise can result in distinct leukocytosis [18, 33]. Thus, it is even more important to avert additional immunosuppression by overexposure to UV radiation. Furthermore, within the PPP group meetings of sample 1, both SAs and coaches identified various barriers toward UVP behavior such as rules for clothing (e.g., no hats for soccer players), set times for midday training, and unavailability of shade. Also, ideas to overcome barriers have been mentioned by coaches. Therefore, in sample 1, we detected a need for support in the motivational but also the volitional phase of establishing UVP behavior in most of the participating SAs and coaches. Less than 50% of the students agreed to the importance of UVP.

Especially, videos and the behavior self-check poster thus regarded the concepts of enhancing self-efficacy, risk perception, and outcome expectancy to support motivational and volitional processes toward a positive attitude for UVP and showing actual UVP behavior. Beside others, theories expect the perception of a certain risk, i.e., for the development of skin cancer, to be necessary to build an intention in favor for a particular health behavior, i.e., sun protection [34]. However, especially for teenagers, appearance motives, self-efficacy, and health-related time perspective seem to be more relevant than risk perception for the intention to avoid the sun [19]. In our study, students required images of the negative consequences of missing UVP like skin cancer or sunburn on the poster. Research is polarized by the effectiveness of fear appeals. But since a recent meta-analysis shows that fear appeals effectively influence attitudes, intentions, and behaviors positively [35], the authors decided to accept this suggestion. Regarding self-identification, only program video ideas that young athletes could identify with were chosen for production. All drafts not primarily linked to the values of athletes reported in the literature but linked to slapstick comedy were less popular.

The reported good reasons, barriers and suggested solutions for sun protection as well as statements regarding content and layout of materials were taken into account in the final materials. All potential future program participants were addressed with targeted UVP messages that were conveyed with the various adapted materials such as videos, posters, and emails.

Ratings on acceptance of and identification with videos and behavior self-check poster of the participating student athletes have been used to modify program material and to adapt it to the needs of this target group. Results of sample 2 show that at least 4 out of 5 students (grade 7, 9, and 12) selected predominantly positive adjectives to describe the videos (80%) and the behavior self-check poster (86%). The adjusted material was mostly described as “important” (videos: 41%; poster: 59%) and “positive” (videos: 31%; poster: 51%). Most of the students reported to identify themselves with the video “For team players,” followed by “For the clever,” and “For the ambitious,” varying with class level. This is in line with the values and attitudes common to athletes, such as being performance oriented and assertive [24–26]. The varying results in different grades indicate that materials could have diverging influences on students of different age groups. This should be addressed in a subsequent study.

With respect to the organizational level, interviews and group discussions with coaches, teachers, and school administration helped to identify aspects that support the implementation of CSSP into school structures and processes. Such aspects comprised offering strategies to handle barriers for UVP in training and competition and recommending wording for integrating UVP into school policy. In addition, it incorporated giving examples on how to integrate UVP into school routine including material to address UVP in class, in parent-teacher conferences, and material to draft a UVP strategy for the school.

In summary, participatory program development may sometimes force the researcher to face some unexpected but difficult decisions, for example, participants proposing the use of fear appeals. Combining top-down and bottom-up processes may impose a challenge. Though, when comparing program materials at step 1 of CSSP development with the completed program kit, CSSP greatly benefited by the PPP approach. Including potential future participants into the process not only enhanced program material but also broadened the program. CSSP now offers material for education on UVP in class, material for application in parent-teacher conferences, in training and competition, as well as material about handling of barriers and the development of a UVP strategy.

### Limitations

This study is limited by the small number of teachers and coaches participating in the PPP process. Because of being involved in class and training, many coaches and teachers canceled PPP group meetings. Parents also have not been part of the PPP process. The sample was not chosen randomly.

The target groups were primarily involved in the development of the concept, content, and materials of CSSP. Concerning PPP component “Program goals,” the target groups could have been involved even more into wordings of goals and objectives. Even though the program takes HAPA into account, the focus of the program is still on promoting positive attitudes toward sun protection among students, teachers, coaches, and parents. By addressing maintenance self-efficacy and recovery self-efficacy, volitional processes could be supported even more. Results of interviews with school administration and of PPP groups may be biased by social desirability. Validity and reliability have not been sufficiently examined. Further stakeholders like the ministries of education and cultural affairs and the *German Olympic Sports Confederation* have not been involved in the development of concept and materials of CSSP.

## Conclusions

In following WHO recommendations for UV protection at schools as well as using participatory program planning, the authors developed the Clever in Sun and Shade Program that aims at enforcing positive attitudes toward UV protection and supporting sports schools in establishing UV-protection strategies. PPP has shown to be an effective and valuable method in constructing CSSP to meet sports schools’ needs. CSSP now consists of a project kit free of charge containing videos with tailored messages targeting students, coaches, teachers, and parents, as well as posters and a manual about UVP and recommendations for implementing UVP in sports school settings.

Summative evaluation that is also founded on the PPP components will be conducted in a subsequent study, followed by program dissemination in cooperation with *German Cancer Aid* and *German Olympic Sports Confederation* (DOSB, *Deutscher Olympischer Sportbund*).

## Data Availability

Upon reasonable request, datasets used during the CSSP development are available from the corresponding author.

## Abbreviations

CSSP: Clever in Sun and Shade Program
DOSB: Deutscher Olympischer Sportbund (German Olympic Sports Confederation)
HAPA: Health action process approach
PPP: Participatory program planning
SA: Student athletes
UV: Ultraviolet
UVP: UV protection
WHO: World Health Organization
